# *Mycoplasma pneumoniae*: Current Knowledge on Nucleic Acid Amplification Techniques and Serological Diagnostics

**DOI:** 10.3389/fmicb.2016.00448

**Published:** 2016-03-31

**Authors:** Katherine Loens, Margareta Ieven

**Affiliations:** Department of Microbiology, National Reference Centre for Respiratory Pathogens, University Hospital AntwerpAntwerp, Belgium

**Keywords:** *Mycoplasma pneumoniae*, serology, nucleic acid amplification test, technological developments

## Abstract

*Mycoplasma pneumoniae* (*M. pneumoniae*) belongs to the class Mollicutes and has been recognized as a common cause of respiratory tract infections (RTIs), including community-acquired pneumonia (CAP), that occur worldwide and in all age groups. In addition, *M. pneumoniae* can simultaneously or sequentially lead to damage in the nervous system and has been associated with a wide variety of other acute and chronic diseases. During the past 10 years, the proportion of LRTI in children and adults, associated with *M. pneumoniae* infection has ranged from 0 to more than 50%. This variation is due to the age and the geographic location of the population examined but also due to the diagnostic methods used. The true role of *M. pneumoniae* in RTIs remains a challenge given the many limitations and lack of standardization of the applied diagnostic tool in most cases, with resultant wide variations in data from different studies. Correct and rapid diagnosis and/or management of *M. pneumoniae* infections is, however, critical to initiate appropriate antibiotic treatment and is nowadays usually done by PCR and/or serology. Several recent reviews, have summarized current methods for the detection and identification of *M. pneumoniae*. This review will therefore provide a look at the general principles, advantages, diagnostic value, and limitations of the most currently used detection techniques for the etiological diagnosis of a *M. pneumoniae* infection as they evolve from research to daily practice.

About 50 years ago, an outbreak of *M. pneumoniae* in a pediatric chronic care facility was described (Baernstein et al., 1965). Twenty years earlier, the organism had been identified by Eaton and since the early 1960s, it was clearly identified as a bacterium which was associated both in children and adults with community-acquired infections of the respiratory tract (Lambert, 1964). Since then, numerous reports have been published on the association of *M. pneumoniae* with community-acquired infections (Waites and Talkington, 2004). Given the wide variations of data from studies with equally wide variation of and lack of standardized diagnostic methods, the true role of *M. pneumoniae* in RTIs still remains a challenge.

Since its discovery, scientists have explored several strategies for an optimal diagnosis of a *M. pneumoniae* infection in the laboratory to initiate an appropriate treatment. Because of its fastidious nature, *M. pneumoniae* is not routinely cultured from respiratory specimens. Culture methods have been the gold standard for diagnosis but are too insensitive producing a result after several days or even several weeks and are therefore not relevant for the management of acute illness. Alternative diagnostic procedures were developed: Detection of IgM and/or IgG by ELISA, antigen detection by immunochromatography, and nucleic acid amplification techniques (NAATs), mainly PCR, although also isothermal amplification techniques such as LAMP (loop-mediated isothermal amplification method) have been developed. The utility of culture for *M. pneumoniae* was assessed by comparing it to PCR and IgM serology in a large study (She et al., 2010). Given the extremely low yield of culture and the wide availability of NAAT and serology, the authors concluded that culture for *M. pneumoniae* should be discontinued. Nowadays, most studies are serology and/or PCR-based. Different clinical specimens can be used as described in the review by Loens et al. (2009) for the latter.

## Application of NAATs

PCR is accepted as a rapid diagnostic test. Few of the currently available NAATs have been extensively validated against culture. The sensitivity of NAATs is almost always superior to that of traditional procedures and they are more and more considered as the “new gold standard.”

An increasing body of literature describing the use of in-house NAATs for detection of *M. pneumoniae* DNA or RNA in various diseases is available with a great variation of methods used from study to study, including variability of target (P1 adhesin gene, 16S rRNA, ATPase gene, protease gene, CARDS toxin gene), NAAT (conventional, nested, real-time; monoplex vs. multiplex; PCR vs. isothermal amplification technologies), detection formats, and different platforms. An overview of the literature on the use of NAATs to detect *M. pneumoniae* since 1989 is given in two reviews (Loens et al., 2003, 2010a).

Lately, efforts have been mainly emphasized on the development of multiplex assays (Nummi et al., 2015; Shen et al., 2015) and on the evaluation of commercially available assays. Respiratory viruses and other so called “atypical bacteria” are all responsible for RTIs that may produce clinically similar manifestations. In order to reduce costs and hands-on-time, multiplex NAATs for the simultaneous detection of 2, 3, or up to more than 20 different respiratory pathogens in one tube with a mixture of primers have been developed by some groups. However, comparison between mono-and multiplex assays has been rarely performed. Findings and conclusions result frequently in contradictory and conflicting data concerning the sensitivity and specificity of the multiplex NAATs compared to the mono NAATs. This is not unexpected since the presence of several pairs of primers may increase the probability of mispairing resulting in non-specific amplification products and the formation of primer-dimers. Furthermore, enzymes, primers, and salt concentrations as well as temperature cyclings required for each target may be slightly different. The results of the proficiency panels (Loens et al., 2010b, 2012) described previously seem to confirm that multiplex assays are somewhat less sensitive than monoplex assays but until the number of organisms present in clinical specimens of diseased individuals is known, it is impossible to state whether the degree of sensitivity attained is clinically acceptable.

Since the previous review (Ieven and Loens, 2013) new NAATs became commercially available such as the Illumigene (Meridian Bioscience, USA) kit. It has been proposed that industry-produced assays in kit form result in better standardization. The analytical sensitivity of the Illumigene assay was evaluated by using 36 frozen stock cultures of *M. pneumoniae* reference strains, and a collection of other microorganisms and human DNA. (Ratliff et al., 2014). Serial dilutions of cultures with a known CFU/ml defined the analytical sensitivity at ≤88 CFU/ml. Based on the results obtained with 214 archived respiratory specimens, previously cultured for *M. pneumoniae*, the clinical sensitivity and specificity were found to be 100 and 99%, respectively, after resolving discrepancies by PCR and sequencing.

A second example of a test approved for the detection of a number of respiratory viruses by the US Food and Drug Administration is the Filmarray Respiratory panel (bioMérieux, France). The Filmarray is a small desktop closed single-piece flow real-time PCR system. It includes automation of nucleic acid extraction, an initial reverse transcription and multiplex PCR, followed by singleplex second stage PCR reactions for the detection of 15 viral agents including adenovirus, coronavirus HKU1, coronavirus NL63, human metapneumovirus, rhinovirus/enterovirus, influenza A/B, influenza A H1, AH1 2009, A H3, parainfluenza 1–4, and respiratory syncytial virus (Poritz et al., 2011). In May 2012, the US Food and Drug Administration expanded the use for the Filmarray respiratory panel with the addition of *B. pertussis, M. pneumonia*, and *C. pneumoniae*. The expanded panel detects now a total of 17 viruses and three bacteria. The test requires 5 min hands-on-time and 65 min instrumentation time. In 2013, a new version of the Filmarray (version 1.7) was released (Doern et al., 2013).

The Argene Respiratory MWSr-gene concept allows the detection of numerous pathogens (Influenza A/B, respiratory syncytial virus/human metapneumovirus, rhinovirus/enterovirus, adenovirus/bocavirus, *Chlamydia/Mycoplasma pneumoniae*, human coronavirus/parainfluenza virus, *Bordetella, Bordetella parapertussis*) in the same run. In addition, the diagnostic strategy can be adapted to the season: searching for the most likely pathogens can be considered in 1st stage, the remaining pathogens being searched for systematically in a 2nd stage.

Pillet et al. (2013) compared six commercially available multiplex assays for the diagnosis of respiratory pathogens. Two out of six were also capable of detecting *M. pneumoniae*: the RespiFinder SMART 22 (PathoFinder, The Netherlands) and the Seeplex RV15 OneStep ACE detection and Pneumobacter ACE detection (Seegene Inc, South Korea). Sensitivities and specificities were calculated against the ArgeneChla/Myco pneumo assay (bioMérieux, France). Sensitivity and specificity were 70.0 and 100%, respectively, for the RespiFinder assay and 80.0 and 98.73% for the Seegene assay.

Dumke et al. compared four commercially available real-time PCR assays recommended for use with the Roche LightCycler 1.5 and 2.0 instruments [Diagenode *Mycoplasma/Chlamydophila pneumoniae* real-time PCR (Diagenode, Belgium), GeneProof *M. pneumoniae* (GeneProof, Czech Republic), BactoReal *M. pneumoniae* (Ingenetix, Austria), LightMix kit *M. pneumoniae* (TIB MOLBIOL, Germany)] for the detection of *M. pneumoniae* to results obtained with an in-house approach (Dumke and Jacobs, 2014) by using serial dilutions of a cultured *M. pneumoniae* strain tested in eight parallel runs and 37 clinical specimens, previously found to be *M. pneumoniae* positive by the in-house assay. All NAATs detected 20 colony forming units (CFU)/5μl sample. Only the in-house-test (repMP1-based approach) was able to detect 0.2 CFU/5μl sample. 37/37,35/37, 35/37, 34/37 *M. pneumoniae* positive clinical specimens were confirmed by the Diagenode test, the Ingenetix and Lightmix assay, and the GeneProof assay respectively.

An overview of commercially available NAATS for the detection of *M. pneumoniae* is presented in Table 1.

**Table 1:** **Summary of commercially available single and multiplex PCR assays for detection of *M. pneumonia***.

**Kit**	**Manufacturer**	**Assay type**	**Detection procedure**	**Pathogens targeted**
*M. pneumoniae* BDProbeTec ET	BD	SDA	Fluorescence	*M. pneumoniae*
ASR MPN	Cepheid	PCR	Real-time	*M. pneumoniae*
Simplex *M. pneumoniae*	Focus diagnostics	PCR	Real-time	*M. pneumoniae*
GeneProof *Mycoplasma pneumoniae*	GeneProof	PCR	Real-time	*M. pneumoniae*
Loopamp *Mycoplasma pneumoniae* DNA amplification kit	Eiken chemical	LAMP	Turbidity	*M. pneumoniae*
BactoReal *Mycoplasma pneumoniae*	Ingenetix	PCR	Real-time	*M. pneumoniae*
**Illumigene *Mycoplasma***	Meridian BioScience	LAMP	Turbidity	*M. pneumoniae*
Venor MP	Minerva BioLabs	PCR	Agarosegel electrophoresis and real-time	*M. pneumoniae*
*M. pneumoniae* LightMix kit	TIB MolBIOL	PCR	Real-time	*M. pneumoniae*
Cp/Mp tracer	Affigene	MX-PCR	Real-time	*M. pneumoniae, C. pneumoniae*
AID CAP bacterial assay	AID GmbH	MX-PCR	ICT	*S. pneumoniae, H. influenzae, M. catarrhalis, C. pneumoniae, M. pneumoniae, L. pneumophila*
Chlamylege	Argene, bioMérieux	MX-PCR	Hybridization	*C. pneumoniae, M. pneumoniae, Legionella spp*
EasyPlex respiratory pathogens B and C	Ausdiagnostics	MX-PCR	Real-time	Influenza A, influenza A H1, influenza A H3, influenza A H5, influenza B, RSV, rhinovirus, enterovirus, PIV 1-3, HAdV, hMPV, HCoV 229E and OC43, *B. pertussis, M. pneumoniae, C. pneumoniae, L. pneumophila, L. longbeachae, Pneumocystis, H. influenzae, S. pneumoniae*
Respiratory Multi Well System Chla/Myco pneumo r-gene	BioMérieux	5 duplex PCRs	Real-time	Influenza A, influenza B, RSV, hBoV, HAdV, hMPV, rhino/enterovirus, *C. pneumoniae, M. pneumoniae*
Diagenode *Mycoplasma/Chlamydophila pneumoniae* real-time PCR kit	Diagenode	Duplex PCR	Real-time	*M. pneumoniae, C. pneumophila*
Fast Track Respiratory Pathogen assay	Fast-track diagnostics	MX-PCR	Real-time	*S. pneumoniae, S. aureus, H. influenzae, M. catarrhalis, Legionella spp, M. pneumoniae, C. pneumoniae*
ProPneumo-1	Hologic	MX-PCR	Real-time	*M. pneumoniae, C. pneumoniae*
ProPneumo1+	Hologic	MX-PCR	Real-time	*M. pneumoniae, C. pneumoniae*
**Filmarray**	BioMérieux.	MX-PCR	Microarray	Influenza A H1N1, influenza A H1, influenza A H3, influenza B, RSV, hMPV, HCoV NL63, OC43, 229E, HKU1, HAdV PIV 1-4, HBoV, rhino/enterovirus, *B. pertussis, M. pneumoniae, C. pneumoniae*
RespiFinder plus	Patho Finder	MX-PCR	Capillary electrophoresis	*M. pneumoniae, C. pneumoniae, L. pneumophila, B. pertussis*, Influenza A (H5, non-specific) and B, RSV A/B, PIV 1-4, rhinovirus, 3 HCoV, hMPV, HAdV
RespiFinder focus	Patho Finder	MX-PCR	Capillary electrophoresis or microfluidics	*M. pneumoniae, C. pneumoniae, L. pneumophila, B. pertussis*, influenza A and B, RSV A/B, hMPV, HAdV
SmartFinder	Patho Finder	MX-PCR	Real-time	*M. pneumoniae, C. pneumoniae, L. pneumophila, B. pertussis*, influenza A and B, RSV A/B, PIV 1-4, HAdV, rhinovirus, 3 HCoV, hMPV, HBoV
Seeplex PneumoBacter ACE	Seegene Inc.	MX-PCR	Capillary electrophoresis	*S. pneumoniae, H. influenzae, M. pneumoniae, C. pneumoniae, L. pneumophila, B. pertussis*
Seeplex RV/PB18 ASE	Seegene Inc.	MX-PCR	Capillary electrophoresis	*S. pneumoniae, H. influenzae, M. pneumoniae, C. pneumoniae, L. pneumophila*, Influenza A and B, RSV A/B, PIV 1-3, rhinovirus, 3 HCoV, HAdV, HBoV, enterovirus

*Bold, FDA-approved test*.

*HAdV, human adenovirus; HBoV, human bocavirus; HCoV, human coronavirus, hMPV, human metapneumovirus; ICT, immunochromatographic test; LAMP, Loop-Mediated isothermal amplification method; MX-PCR, multiplex PCR; PIV, parainfluenzavirus; qPCR, quantitative PCR*.

Since the calculation of the sensitivities of the commercial multiplex assays was mainly dependent on DNA copy number, further evaluation and standardization using an extended number of clinical specimens that may have a low bacterial load are needed. The use of an international standard developed by the WHO for harmonization of *Mycoplasma* NAAT (Nübling et al., 2015) or the yearly participation in the external quality assessment (EQA) panel for *M. pneumoniae* and *Chlamydophila pneumoniae* available from Quality Control for Molecular Diagnostics (QCMD, United Kingdom) should be considered.

So far, it is unclear whether asymptomatic carriage of *M. pneumoniae* in adults and children exists and if colonization could be differentiated from infection by the current diagnostic methods. There are only few data on the relation between the bacterial load and the severity of infection. 405 asymptomatic children and 321 children with a RTI were enrolled in a cross-sectional study (Spuesens et al., 2013). Nasopharyngeal washings and pharyngeal swabs were investigated by culture and quantitative real-time PCR (qPCR). Serum was collected for IgM and IgG ELISA. Neither qPCR, serology nor culture was capable of differentiating colonization from infection. In 21.2 and 16.2% of the asymptomatic and symptomatic children, *M. pneumoniae* DNA was detected. In addition, persistence of *M. pneumoniae* in the upper respiratory tract was shown for up to 4 months by longitudinal sampling. A retrospective study investigated the clinical significance of the *M. pneumoniae* bacterial load in children with a *M. pneumoniae* pneumonia (Jiang et al., 2014). The authors concluded that a high bacterial load was indicative for a *M. pneumoniae* infection, whereas for a low bacterial load the etiologic role of *M. pneumoniae* remains to be determined.

Edin et al. developed a qPCR with duplex reactions targeting eight bacteria, including *M. pneumoniae*, and six viruses (Edin et al., 2015). Clinical specimens from the upper and lower respiratory tract were used to compare the qPCR assay with standard microbiological methods. The use of the qPCR assay resulted in 113 positive identifications in 94 respiratory specimens compared with 38 by using standard diagnostics. The authors conclude that in parallel qPCR detection of the targeted respiratory bacteria and viruses is feasible since a good technical performance of the assay in clinical specimens was obtained.

In contrast to the above mentioned studies, Jain et al. (2015) examined specimens from 2222 hospitalized children with community-acquired pneumonia and 521 asymptomatic controls for the detection of a variety of respiratory pathogens. *M. pneumoniae* was detected in 8%, and in 3% or less of controls.

Another trend is the simultaneous detection of *M. pneumoniae* and mutations associated with macrolide resistance directly in clinical specimens (Ji et al., 2014; Liu et al., 2014; Nummi et al., 2015; Zhao et al., 2015).

## Application of serology for the detection of *M. pneumoniae* infections

Serological methods, in particular enzyme-linked immunosorbent assays (ELISA), are most widely used to diagnose a *M. pneumoniae* infection. The complement fixation test (CFT) has been replaced by assays which allow for quantification of IgM, IgA, or IgG. However, the most convincing evidence of an ongoing infection is a significant increase in IgG or an IgG seroconversion in paired sera, collected 3–4 weeks apart (Nir-Paz et al., 2006). Although IgM antibodies appear earlier than IgG antibodies, and are thus an attractive alternative for diagnosis of a *M. pneumoniae* infection, one should realize that IgM is not often produced in very young children, in a proportion of primary infections and during re-infections (Waites et al., 2008; Loens et al., 2010a).

Ten serological assays for the diagnosis of a *M. pneumoniae* infection were recently evaluated by using 145 sera from 120 patients (Busson et al., 2013): SeroMP IgM and IgG (Savyon Diagnostics), SeroMP Recombinant IgM, IgA and IgG (Savyon Diagnostics), LIAISON *M. pneumoniae* IgM and IgG (Biotrin International Ltd), *M. pneumoniae* IgM, IgA and IgG Medac (Medac GmbH). A low IgM specificity and cross-reactivity was noticed for the SeroMP recombinant and Liaison assay. For IgA, the Medac assay tended to be less specific than the SeroMP Recombinant assay. All four tests showed discrepancies in the IgG measurements confirming results of previous studies (Talkington et al., 2004; Beersma et al., 2005). In conclusion, serology remains a diagnostic tool of choice but improvement and standardization of the assays are still needed, especially for the determination of IgG.

The clinical significance of a serologic test, both for IgM and IgG, should be defined by studies of patients with a documented infection and for whom detailed information concerning the time lapses between onset of disease and the collection of the serum specimens are known.

A promising blotting technique improving the performance of the *M. pneumoniae* serological assays has been described (Dumke et al., 2012).

## Detection of *M. pneumoniae* by both NAATs and serology

Data from recent studies using PCR based methods and serology published during the last decade in different patient populations from around the world are summarized in the recent reviews published by Ieven and Loens (Loens et al., 2010a; Ieven and Loens, 2013) and updated in Table 2.

Table 2**Summary of recent single and multiplex NAATs for detection of *Mycoplasma pneumoniae* published since the previous review, and previously validated assays used as comparators**.

**Table:** 

**Monoplex assays**						
**Assay year (references)**	**Assay type**	**Detection format**	**Gene target (product size)**	**PCR assay used as comparator for new assay**	**Non-PCR comparator test**	**Specimens tested for validation of sensitivity and or specificity**
2012 (Zhao et al., 2012)	PCR	Real-time	P1-gene (72)	repMp1 and Mp181		Various bacterial species, bacterial dilution series, well-defined clinical specimens
2012 (Gotoh et al., 2012)	LAMP	Turbidity	P1 operon (NS) (Eiken Chemical)		IgG seroconversion/significant rise	Samples from 368 pneumonia patients
2013 (Chaudhry et al., 2013)	PCR	Real-time	P1-gene (534)	Conventional PCR (NS)	IgM, IgG and IgA serology	Dilution series, respiratory samples from CAP-patients,
2013 (Schmitt et al., 2013)	PCR	Real-time	ptsI (160)	LightMix kit *M. pneumoniae* (TIB MOLBIO), *M. pneumoniae* analyte specific reagent (Focus diagnostics), (Dumke et al., 2007)		Bacterial dilution series, spiked clinical specimens, well-defined clinical specimens
2014 (Liu et al., 2014)	PCR	Cycleave	23S rDNA (Takara BioInc)	(Ieven et al., 1996)		Various bacterial species, bacterial dilution series, clinical specimens
2014 (Ratliff et al., 2014)	LAMP	Turbidity	Illumigene assay (208)	2nd real-time PCR and sequencing	Culture	Various bacterial species, bacterial dilution series, 214 culture positive/negative specimens
2014 (Medjo et al., 2014)	PCR	Real-time	P1 (125)		IgM and IgG serology, culture	Specimens from CAP-patients

**Table:** 

**Multiplex assays**						
**Assay year (references)**	**Assay type**	**Detection format**	**Specimens tested for validation of sensitivity and or specificity**
2013 (Puppe et al., 2013)	MX-PCR	ELISA	Culture supernatans of the organisms, clinical specimens from frozen stocks, prospectively included nasopharyngeal aspirates
			Pathogens targeted: enterovirus, influenza A, influenza B, RSV, PIV 1-4, HAdV, rhinovirus, hMPV, HCoV, reovirus, *M. pneumoniae, C. pneumoniae, B. pertussis, B. parapertussis, L. pneumophila*
2013 (Simões et al., 2013)	MX-PCR	Affimetrix Chip-image file	Clinical specimens simultaneously investigated by culture and two commercially available assays: the Eragen assay and the Luminex RVP
			Pathogens targeted: 72 pathogens
2013 (Weinberg et al., 2013)	MX-PCR	TAC-array	Well-defined clinical specimens analyzed by individual real-time PCRs
			Pathogens targeted: HAdV, hMPV, PIV1-4, influenza A, influenza B, influenza C, RSV, rhinovirus, HCoV OC43, 229E, NL63, HKU1, enterovirus, *B. pertussis, C. pneumoniae, H. influenza, L. pneumophila, M. pneumoniae, S. pneumoniae, S. pyogenes*
2014 (Hirama et al., 2014)	MX-PCR	Real-time	DNA dilution series, welldefined clinical specimens from CAP-patients
			Pathogens targeted: *S. pneumoniae, H. influenza, M. catarrhalis, P. aeruginosa, K. pneumoniae, E. coli, S. aureus, M. pneumoniae, C. pneumoniae, C. psittaci, C. burnetii, Legionella spp, L. pneumophila, B. pertussis, M. tuberculosis, M. intracellulare, M. avium, M. kansasii, P. jeroveci, Nocardia spp*, metallo-beta-lactamase, MRSA
2014 (Ji et al., 2014)	MX-PCR	Agarose gel electrophoresis	Various bacterial pathogens, bacterial dilution series, confirmation by sequencing, well-defined clinical specimens
			Pathogens targeted: *M. pneumoniae* and associated macrolide resistance
2015 (Zhao et al., 2015)	Duplex PCR	Real-time	Pathogens targeted: *M. pneumoniae* and genotyping
2015 (Shen et al., 2015)	MX-PCR	Resequencing microarray	Pathogens targeted: *S. pneumoniae, M. pneumoniae, H. influenza, K. pneumoniae, M. catarrhalis, S. aureus, P. aeruginosa, M. tuberculosis, N. meningitidis*, Group A Streptococci
2015 (Nummi et al., 2015)	MX-PCR	Real-time	Pathogens targeted: *M. pneumoniae, C. pneumoniae* and mutations associated with macrolide resistance

HAdV, human adenovirus; HBoV, human bocavirus; HCoV, human coronavirus; hMPV, human metapneumovirus; PIV, parainfluenzavirus; MX-PCR, multiplex PCR; LAMP, Loop-mediated isothermal amplification; TAC, Taqman Array Card.

The availability of the very sensitive NAATs has in recent years also put the often used serological tests in their right perspective and allow a better interpretation of the serological test results and their limitations such as the low sensitivity of IgM antibodies in acute phase specimens and importance of the delay between two serum samples. Studies in which also NAAT's are used on respiratory specimens should allow a better interpretation of the serological test results.

A rapid response report from the Canadian Agency for Drugs and Technologies in Health (Canadian Agency for Drugs and Technologies in Health, 2015) presents the results of a literature search in order to identify the diagnostic test accuracy, clinical effectiveness, and cost-effectiveness of serum IgM and molecular tests for the detection of *M. pneumoniae* in patients with a respiratory infection^1^. Six relevant studies were identified, but no evidence regarding the clinical effectiveness or cost-effectiveness of a serum IgM test compared with molecular tests was identified. Zhang et al. conducted a systematic review and meta-analysis on the diagnosis of *M. pneumoniae* by PCR and serology (Zhang et al., 2011) and reported a significant heterogeneity between the studies and inconsistent results as well.

Two studies compared the application of real-time PCR and serology in children with pneumonia. In 2011, 54/290 children were found to be positive by PCR (Chang et al., 2014), 44/182 were *M. pneumoniae* IgM positive. 12.6% of patients were found to be *M. pneumoniae* positive by both tests at the same time. Using PCR as gold standard, a sensitivity and specificity of resp. 62.2 and 85.5% were obtained. The specificity could be increased to 90.3% by increasing the cut-off without changing the sensitivity of the IgM assay. A study conducted by Medjo et al. (2014) applied PCR, culture, IgM and IgG in paired sera for the detection of a *M. pneumoniae* infection in 166 children. Using IgG serology as gold standard, the sensitivity of IgM, PCR, and culture was found to be equal (81.8%), specificity was found to be 100, 98.6, and 100% respectively. It was concluded that during the acute phase of disease, detection of IgM antibodies in combination with PCR allowed for a precise and reliable *M. pneumoniae* diagnosis. A prospective study in children with community-acquired CAP (Kakuya et al., 2014) compared loop-mediated isothermal amplification, (LAMP), culture and serology at first visit. Patients were defined positive if positive by culture and/or sero-conversion or a four-fold increase in IgG in paired sera. 31/191 patients met the criteria. Thirteen were positive by culture and serology, 17 on culture only, and one by serology only. A positive LAMP result was obtained for all patients that were culture positive. The sensitivity and specificity for LAMP, EIA, and the particle agglutination test, were 96.8, 38.7, 19.4, and 100%, 76.9 and 93.1%, respectively.

When establishing the etiology in 267 adult CAP-patients in Norway, 10 were found to be *M. pneumoniae* positive: two by serology, seven and one by PCR applied to a nasopharyngeal flocked swab and an oropharyngeal flocked swab, respectively (Holter et al., 2015).

## Amplification-free and other technological developments

Newer technologies such as microfluidics and the application of nanotechnology offer the potential to an even more rapid detection of important pathogens allowing even near-patient testing. Since these technologies, as NAATs, do not require viable organisms, and thus avoid any adverse effect of longer specimen transport, they can be successfully applied to both the in- and outpatient settings. Several companies currently possess the technical expertise and research infrastructure to bring a useful diagnostic testing approach to the clinical trial stage shortly.

Li et al. (2015) developed a colloidal gold-based immune-chromatographic assay by using a pair of monoclonal antibodies targeting a region of the P1 gene. When applied to 303 clinical specimens from children suspected with a *M. pneumoniae* infection, the sensitivity and specificity against real-time PCR were 100 and 97.4%. This is in contrast to the results obtained with a commercially available rapid antigen test targeting the ribosomal protein L7/L12 (Ribotest Mycoplasma). Compared to real-time PCR, a sensitivity and specificity of respectively 62.5 and 90.9% were obtained when applied to clinical specimens (Miyashita et al., 2015). Based on these results, the authors concluded that treatment decisions should not be taken based on the Ribotest results alone.

Other amplification-free detection methodologies are currently being developed as biosensing detection strategies: A proto-type of an enzyme-free electrochemical genosensor on nanostructured screen-printed gold electrodes (Garcia-Gonzalez et al., 2015); A silver nanorod array-surface enhanced Raman Spectroscopy biosensing platform was successfully applied for the detection of *M. pneumoniae* in simulated and clinical throat swabs (Henderson et al., 2014, 2015).

## Conclusions

With the use of tools such as NAATs a greater understanding of the etiology and epidemiology of *M. pneumoniae* is possible. Taken into account the results obtained in recent studies, there is more evidence that real-time NAATs are superior to other *M. pneumoniae* detection strategies during the early phase of infection. NAATs, however, cannot completely replace serology. In epidemiological studies, serology is certainly more useful than for the management of individual patients with LRTI or even CAP since results are often delayed by the need for paired sera to detect a seroconversion or a significant rise in titer; early in the course of an infection, false-negative results often occur.

In case a specific IgM test is used, serology should not completely be abolished despite the fact that IgM serology shows a moderate sensitivity. Nowadays, a combination of the detection of IgM antibodies and PCR may be the most optimal approach for early diagnosis of a *M. pneumoniae* infection, especially in children.

The implementation of quantitative tests could shed further light on the relation between bacterial load and the seriousness of the disease, produce useful prognostic information and help in the differentiation between colonization and infection. More information could be gathered on the length of the post infection carrier state as well as on the importance of subclinical infections and how prone these are for spreading infection.

It remains important to recognize the urgent need for the adoption of a more unified and consistent diagnostic approach for current and future investigations. Therefore, a common set of recommendations should be developed.

## Author contributions

KL drafted the manuscript. GI revised and approved the final manuscript.

### Conflict of interest statement

The authors declare that the research was conducted in the absence of any commercial or financial relationships that could be construed as a potential conflict of interest.

## Footnotes

^1^(2015). Serum IgM and Molecular Tests for Mycoplasma pneumoniae Detection: A Review of Diagnostic Test Accuracy, Clinical Effectiveness, Cost-Effectiveness, and Guidelines, Ottawa, ON.

## References

[B1] Baernstein H. D. Jr. Trevisani E. Axtell S. Quilligan J. J. Jr (1965). *Mycoplasma pneumoniae* (eaton atypical pneumonia agent) in children's respiratory infections. J. Pediatr. 66, 829–837. 10.1016/S0022-3476(65)80057-9 14279842

[B2] Beersma M. F. Dirven K. van Dam A. P. Templeton K. E. Claas E. C. Goossens H. (2005). Evaluation of 12 commercial tests and the complement fixation test for *Mycoplasma pneumoniae*-specific immunoglobulin G (IgG) and IgM antibodies, with PCR used as the “gold standard.” J. Clin. Microbiol. 43, 2277–2285. 10.1128/JCM.43.5.2277-2285.2005 15872256 PMC1153783

[B3] Busson L. Van den Wijngaert S. Dahma H. Decolvenaer M. Di Cesare L. Martin A.. (2013). Evaluation of 10 serological assays for diagnosing *Mycoplasma pneumoniae* infection. Diagn. Microbiol. Infect. Dis. 76, 133–137. 10.1016/j.diagmicrobio.2013.02.027 23537789 PMC7127255

[B4] Canadian Agency for Drugs Technologies in Health (2015). Serum IgM and Molecular Tests for Mycoplasma pneumoniae Detection: A Review of Diagnostic Test Accuracy, Clinical Effectiveness, Cost-Effectiveness, and Guidelines [Internet]. CADTH Rapid Response Reports. Ottawa, ON: Canadian Agency for Drugs and Technologies in Health. 26677498

[B5] Chang H. Y. Chang L. Y. Shao P. L. Lee P. I. Chen J. M. Lee C. Y.. (2014). Comparison of real-time polymerase chain reaction and serological tests for the confirmation of *Mycoplasma pneumoniae* infection in children with clinical diagnosis of atypical pneumonia. J. Microbiol. Immunol. Infect. 47, 137–144. 10.1016/j.jmii.2013.03.015 23726653

[B6] Chaudhry R. Sharma S. Javed S. Passi K. Dey A. B. Malhotra P. (2013). Molecular detection of *Mycoplasma pneumoniae* by quantitative real-time PCR in patients with community acquired pneumonia. Indian J.Med. Res. 138, 244–251. 24056602 PMC3788211

[B7] Doern C. D. Lacey D. Huang R. Haag C. (2013). Evaluation and implementation of FilmArray version 1.7 for improved detection of adenovirus respiratory tract infection. J. Clin. Microbiol. 51, 4036–4039. 10.1128/JCM.02546-13 24068007 PMC3838020

[B8] Dumke R. Jacobs E. (2014). Evaluation of five real-time PCR assays for detection of *Mycoplasma pneumoniae*. J. Clin. Microbiol. 52, 4078–4081. 10.1128/JCM.02048-14 25210063 PMC4313229

[B9] Dumke R. Schurwanz N. Lenz M. Schuppler M. Lück C. Jacobs E. (2007). Sensitive detection of *Mycoplasma pneumoniae* in human respiratory tract samples by optimized real-time PCR approach. J. Clin. Microbiol. 45, 2726–2730. 10.1128/JCM.00321-07 17537933 PMC1951254

[B10] Dumke R. Strubel A. Cyncynatus C. Nuyttens H. Herrmann R. Lück C.. (2012). Optimized serodiagnosis of *Mycoplasma pneumoniae* infections. Diagn. Microbiol. Infect. Dis. 73, 200–203. 10.1016/j.diagmicrobio.2012.02.014 22502960

[B11] Edin A. Granholm S. Koskiniemi S. Allard A. Sjöstedt A. Johansson A. (2015). Development and laboratory evaluation of a real-time PCR assay for detecting viruses and bacteria of relevance for community-acquired pneumonia. J. Mol. Diagn. 17, 315–324. 10.1016/j.jmoldx.2015.01.005 25772704 PMC7185852

[B12] García-González R. Costa-García A. Fernández-Abedul M. T. (2015). Enzymatic amplification-free nucleic acid hybridisation sensing on nanostructured thick-film electrodes by using covalently attached methylene blue. Talanta 142, 11–19. 10.1016/j.talanta.2015.03.028 26003686

[B13] Gotoh K. Nishimura N. Ohshima Y. Arakawa Y. Hosono H. Yamamoto Y.. (2012). Detection of *Mycoplasma pneumoniae* by loop-mediated isothermal amplification (LAMP) assay and serology in pediatric community-acquired pneumonia. J. Infect. Chemother. 18, 662–667. 10.1007/s10156-012-0388-5 22370920

[B14] Henderson K. C. Benitez A. J. Ratliff A. E. Crabb D. M. Sheppard E. S. Winchell J. M.. (2015). Specificity and strain-typing capabilities of nanorod array-surface enhanced raman spectroscopy for *Mycoplasma pneumoniae* detection. PLoS ONE 10:e0131831. 10.1371/journal.pone.0131831 26121242 PMC4487258

[B15] Henderson K. C. Sheppard E. S. Rivera-Betancourt O. E. Choi J. Y. Dluhy R. A. Thurman K. A.. (2014). The multivariate detection limit for *Mycoplasma pneumoniae* as determined by nanorod array-surface enhanced Raman spectroscopy and comparison with limit of detection by qPCR. Analyst 139, 6426–6434. 10.1039/C4AN01141D 25335653 PMC4329772

[B16] Hirama T. Minezaki S. Yamaguchi T. Kishi E. Kodama K. Egashira H.. (2014). HIRA-TAN: a real-time PCR-based system for the rapid identification of causative agents in pneumonia. Respir. Med. 108, 395–404. 10.1016/j.rmed.2013.11.018 24411834

[B17] Holter J. C. Müller F. Bjørang O. Samdal H. H. Marthinsen J. B. Jenum P. A.. (2015). Etiology of community-acquired pneumonia and diagnostic yields of microbiological methods: a 3-year prospective study in Norway. BMC Infect. Dis. 15:64. 10.1186/s12879-015-0803-5 25887603 PMC4334764

[B18] Ieven M. Loens K. (2013). Should serology be abolished in favor of PCR for the diagnosis of *Mycoplasma pneumoniae* infections? Curr. Pediatr. Rev. 9, 304–313. 10.2174/157339630904131223110501

[B19] Ieven M. Ursi D. Van Bever H. Quint W. Niesters H. G. Goossens H. (1996). Detection of *Mycoplasma pneumoniae* by two polymerase chain reactions and role of *M. pneumoniae* in acute respiratory tract infections in pediatric patients. J. Infect. Dis. 173, 1445–1452. 10.1093/infdis/173.6.1445 8648218

[B20] Jain S. Williams D. J. Arnold S. R. Ampofo K. Bramley A. M. Reed C.. (2015). Community-acquired pneumonia requiring hospitalization among U.S. children. N. Engl. J. Med. 372, 835–845. 10.1056/NEJMoa1405870 25714161 PMC4697461

[B21] Ji M. Lee N. S. Oh J. M. Jo J. Y. Choi E. H. Yoo S. J.. (2014). Single-nucleotide polymorphism PCR for the detection of *Mycoplasma pneumoniae* and determination of macrolide resistance in respiratory samples. J. Microbiol. Methods 102, 32–36. 10.1016/j.mimet.2014.04.009 24780151

[B22] Jiang W. Yan Y. Ji W. Wang Y. Chen Z. (2014). Clinical significance of different bacterial load of *Mycoplasma pneumoniae* in patients with *Mycoplasma pneumoniae* pneumonia. Braz. J. Infect. Dis. 18, 124–128. 10.1016/j.bjid.2013.06.004 24650994 PMC9427492

[B23] Kakuya F. Kinebuchi T. Fujiyasu H. Tanaka R. Kano H. (2014). Genetic point-of-care diagnosis of *Mycoplasma pneumoniae* infection using LAMP assay. Pediatr. Int. 56, 547–552. 10.1111/ped.12327 24612134

[B24] Lambert H. P. (1964). Eaton agent and other non-bacterial pneumonias. Public Health 78, 335–341. 10.1016/S0033-3506(64)80050-0 14199665

[B25] Li W. Liu Y. Zhao Y. Tao R. Li Y. Shang S. (2015). Rapid diagnosis of *Mycoplasma pneumoniae* in children with pneumonia by an immuno-chromatographic antigen assay. Sci. Rep. 5:15539. 10.1038/srep15539 26486047 PMC4614389

[B26] Liu Y. Ye X. Zhang H. Wu Z. Xu X. (2014). Rapid detection of *Mycoplasma pneumoniae* and its macrolide-resistance mutation by Cycleave PCR. Diagn. Microbiol. Infect. Dis. 78, 333–337. 10.1016/j.diagmicrobio.2013.12.002 24503503

[B27] Loens K. Goossens H. Ieven M. (2010a). Acute respiratory infection due to *Mycoplasma pneumoniae*: current status of diagnostic methods. Eur. J. Clin. Microbiol. Infect. Dis. 29, 1055–1069. 10.1007/s10096-010-0975-2 20526788 PMC7088226

[B28] Loens K. Mackay W. G. Scott C. Goossens H. Wallace P. Ieven M. (2010b). A multicenter pilot external quality assessment programme to assess the quality of molecular detection of *Chlamydophila pneumoniae* and *Mycoplasma pneumoniae*. J. Microbiol. Methods 82, 131–135. 10.1016/j.mimet.2010.05.006 20493214

[B29] Loens K. Ursi D. Goossens H. Ieven M. (2003). Molecular diagnosis of *Mycoplasma pneumoniae* respiratory tract infections. J. Clin. Microbiol. 41, 4915–4923. 10.1128/JCM.41.11.4915-4923.2003 14605118 PMC262541

[B30] Loens K. Van Heirstraeten L. Malhotra-Kumar S. Goossens H. Ieven M. (2009). Optimal sampling sites and methods for detection of pathogens possibly causing community-acquired lower respiratory tract infections. J. Clin. Microbiol. 47, 21–31. 10.1128/JCM.02037-08 19020070 PMC2620840

[B31] Loens K. van Loon A. M. Coenjaerts F. van Aarle Y. Goossens H. Wallace P.. (2012). Performance of different mono- and multiplex nucleic acid amplification tests on a multipathogen external quality assessment panel. J. Clin. Microbiol. 50, 977–987. 10.1128/JCM.00200-11 22170925 PMC3295136

[B32] Medjo B. Atanaskovic-Markovic M. Radic S. Nikolic D. Lukac M. Djukic S. (2014). *Mycoplasma pneumoniae* as a causative agent of community-acquired pneumonia in children: clinical features and laboratory diagnosis. Ital. J. Pediatr. 40:104. 10.1186/s13052-014-0104-4 25518734 PMC4279889

[B33] Miyashita N. Kawai Y. Tanaka T. Akaike H. Teranishi H. Wakabayashi T.. (2015). Diagnostic sensitivity of a rapid antigen test for the detection of *Mycoplasma pneumoniae*: comparison with real-time PCR. J. Infect. Chemother. 21, 473–475. 10.1016/j.jiac.2015.02.007 25818195

[B34] Nir-Paz R. Michael-Gayego A. Ron M. Block C. (2006). Evaluation of eight commercial tests for *Mycoplasma pneumoniae* antibodies in the absence of acute infection. Clin. Microbiol. Infect. 12, 685–688. 10.1111/j.1469-0691.2006.01469.x 16774570

[B35] Nübling C. M. Baylis S. A. Hanschmann K. M. Montag-Lessing T. Chudy M. Kress J.. (2015). World health organization international standard to harmonize assays for detection of mycoplasma DNA. Appl. Environ. Microbiol. 81, 5694–5702. 10.1128/AEM.01150-15 26070671 PMC4551258

[B36] Nummi M. Mannonen L. Puolakkainen M. (2015). Development of a multiplex real-time PCR assay for detection of *Mycoplasma pneumoniae, Chlamydia pneumoniae* and mutations associated with macrolide resistance in *Mycoplasma pneumoniae* from respiratory clinical specimens. Springerplus 4:684. 10.1186/s40064-015-1457-x 26576327 PMC4641141

[B37] Pillet S. Lardeux M. Dina J. Grattard F. Verhoeven P. Le Goff J.. (2013). Comparative evaluation of six commercialized multiplex PCR kits for the diagnosis of respiratory infections. PLoS ONE 8:e72174. 10.1371/journal.pone.0072174 24058410 PMC3751960

[B38] Poritz M. A. Blaschke A. J. Byington C. L. Meyers L. Nilsson K. Jones D. E.. (2011). FilmArray, an automated nested multiplex PCR system for multi-pathogen detection: development and application to respiratory tract infection. PLoS ONE 6:e26047. 10.1371/journal.pone.0026047 22039434 PMC3198457

[B39] Puppe W. Weigl J. Gröndahl B. Knuf M. Rockahr S. von B. P.. (2013). Validation of a multiplex reverse transcriptase PCR ELISA for the detection of 19 respiratory tract pathogens. Infection 41, 77–91. 10.1007/s15010-012-0298-6 22847627 PMC7100787

[B40] Ratliff A. E. Duffy L. B. Waites K. B. (2014). Comparison of the illumigene Mycoplasma DNA amplification assay and culture for detection of *Mycoplasma pneumoniae*. J. Clin. Microbiol. 52, 1060–1063. 10.1128/JCM.02913-13 24430454 PMC3993520

[B41] Schmitt B. H. Sloan L. M. Patel R. (2013). Real-time PCR detection of *Mycoplasma pneumoniae* in respiratory specimens. Diagn. Microbiol. Infect. Dis. 77, 202–205. 10.1016/j.diagmicrobio.2013.07.016 24041553

[B42] She R. C. Thurber A. Hymas W. C. Stevenson J. Langer J. Litwin C. M.. (2010). Limited utility of culture for *Mycoplasma pneumoniae* and *Chlamydophila pneumoniae* for diagnosis of respiratory tract infections. J. Clin. Microbiol. 48, 3380–3382. 10.1128/JCM.00321-10 20610673 PMC2937689

[B43] Shen H. Zhu B. Wang S. Mo H. Wang J. Li J.. (2015). Association of targeted multiplex PCR with resequencing microarray for the detection of multiple respiratory pathogens. Front. Microbiol. 6:532. 10.3389/fmicb.2015.00532 26074910 PMC4446546

[B44] Simões E. A. Patel C. Sung W. K. Lee C. W. Loh K. H. Lucero M.. (2013). Pathogen chip for respiratory tract infections. J. Clin. Microbiol. 51, 945–953. 10.1128/JCM.02317-12 23303493 PMC3592061

[B45] Spuesens E. B. Fraaij P. L. Visser E. G. Hoogenboezem T. Hop W. C. van Adrichem L. N.. (2013). Carriage of *Mycoplasma pneumoniae* in the upper respiratory tract of symptomatic and asymptomatic children: an observational study. PLoS Med. 10:e1001444. 10.1371/journal.pmed.1001444 23690754 PMC3653782

[B46] Talkington D. F. Shott S. Fallon M. T. Schwartz S. B. Thacker W. L. (2004). Analysis of eight commercial enzyme immunoassay tests for detection of antibodies to *Mycoplasma pneumoniae* in human serum. Clin. Diagn. Lab Immunol. 11, 862–867. 10.1128/cdli.11.5.862-867.2004 15358644 PMC515281

[B47] Waites K. B. Balish M. F. Atkinson T. P. (2008). New insights into the pathogenesis and detection of *Mycoplasma pneumoniae* infections. Future Microbiol. 3, 635–648. 10.2217/17460913.3.6.635 19072181 PMC2633477

[B48] Waites K. B. Talkington D. F. (2004). *Mycoplasma pneumoniae* and its role as a human pathogen. Clin. Microbiol. Rev. 17, 697–728. 10.1128/CMR.17.4.697-728.2004 15489344 PMC523564

[B49] Weinberg G. A. Schnabel K. C. Erdman D. D. Prill M. M. Iwane M. K. Shelley L. M.. (2013). Field evaluation of TaqMan Array Card (TAC) for the simultaneous detection of multiple respiratory viruses in children with acute respiratory infection. J. Clin. Virol. 57, 254–260. 10.1016/j.jcv.2013.03.016 23608639 PMC7108303

[B50] Zhang L. Zong Z. Y. Liu Y. B. Ye H. Lv X. J. (2011). PCR versus serology for diagnosing *Mycoplasma pneumoniae* infection: a systematic review & meta-analysis. Indian J. Med. Res. 134, 270–280. 21985809 PMC3193707

[B51] Zhao F. Cao B. He L. H. Yin Y. D. Tao X. X. Song S. F.. (2012). Evaluation of a new real-time PCR assay for detection of *Mycoplasma pneumoniae* in clinical specimens. Biomed. Environ. Sci. 25, 77–81. 10.3967/0895-3988.2012.01.011 22424630

[B52] Zhao F. Liu L. Tao X. He L. Meng F. Zhang J. (2015). Culture-independent detection and genotyping of *Mycoplasma pneumoniae* in clinical specimens from Beijing, China. PLoS ONE 10:e0141702. 10.1371/journal.pone.0141702 26509651 PMC4625007

